# Comparative Transcriptome Analysis Provides Insights into the Seed Germination in Cotton in Response to Chilling Stress

**DOI:** 10.3390/ijms21062067

**Published:** 2020-03-18

**Authors:** Qian Shen, Siping Zhang, Shaodong Liu, Jing Chen, Huijuan Ma, Ziqian Cui, Xiaomeng Zhang, Changwei Ge, Ruihua Liu, Yang Li, Xinhua Zhao, Guozheng Yang, Meizhen Song, Chaoyou Pang

**Affiliations:** 1 State Key Laboratory of Cotton Biology, Institute of Cotton Research of CAAS, Anyang, Henan 455000, China; shenqian429@126.com (Q.S.); zhsp5337@163.com (S.Z.); mrliusd@163.com (S.L.); chenjing123sky@163.com (J.C.); binghan1201@163.com (H.M.); czq1165857274@163.com (Z.C.); 18738237057@163.com (X.Z.); zhugechangwei@126.com (C.G.); zmslrh@163.com (R.L.); liyangzmr@163.com (Y.L.); zhaoxinhua1968@126.com (X.Z.); 2 MOA Key Laboratory of Crop Eco-physiology and Farming system in the Middle Reaches of Yangtze River, College of Plant Science and Technology, Huazhong Agricultural University, Wuhan 430000, China

**Keywords:** cotton (*Gossypium hirsutum* L.), chilling stress, germination, RNA-Seq, differentially expressed genes

## Abstract

*Gossypium hirsutum* L., is a widely cultivated cotton species around the world, but its production is seriously threatened by its susceptibility to chilling stress. Low temperature affects its germination, and the underlying molecular mechanisms are rarely known, particularly from a transcriptional perspective. In this study, transcriptomic profiles were analyzed and compared between two cotton varieties, the cold-tolerant variety KN27-3 and susceptible variety XLZ38. A total of 7535 differentially expressed genes (DEGs) were identified. Among them, the transcripts involved in energy metabolism were significantly enriched during germination based on analysis of Kyoto Encyclopedia of Genes and Genomes (KEGG) pathways, such as glycolysis/gluconeogenesis, tricarboxylic acid cycle (TCA cycle), and glyoxylate cycle (GAC). Results from further GO enrichment analysis show the earlier appearance of DNA integration, meristem growth, cotyledon morphogenesis, and other biological processes in KN27-3 compared with XLZ38 under chilling conditions. The synthesis of asparagine, GDP-mannose, and trehalose and the catabolic process of raffinose were activated. DEGs encoding antioxidants (spermidine) and antioxidase (CAT1, GPX4, DHAR2, and APX1) were much more up-regulated in embryos of KN27-3. The content of auxin (IAA), cis-zeatin riboside (cZR), and trans-zeatin riboside (tZR) in KN27-3 are higher than that in XLZ38 at five stages (from 12 h to 54 h). GA3 was expressed at a higher level in KN27-3 from 18 h to 54 h post imbibition compared to that in XLZ38. And abscisic acid (ABA) content of KN27-3 is lower than that in XLZ38 at five stages. Results from hormone content measurements and the related gene expression analysis indicated that IAA, CTK, and GA3 may promote germination of the cold-tolerant variety, while ABA inhibits it. These results expand the understanding of cottonseed germination and physiological regulations under chilling conditions by multiple pathways.

## 1. Introduction

Cotton (*Gossypium hirsutum* L.; AADD, 2n = 4× = 52) is the world’s most important crop for natural fiber, but it is sensitive to chilling stress [1]. With the increasing needs of cotton, the cotton planting regions have expanded into the areas of high latitude and altitude with unsuitable climates [2]. Planting normally starts at spring when the night temperatures are well below 12 °C for most cotton-growing regions, including southeastern Turkey [3] and northwestern China [4]. Low temperature leads to delayed growth and development, especially at the germination and seedling stages [5]. In the meantime, more frequent chilling stress further affects cotton production [6].

Plants respond to low temperature with a series of physiological and biochemical reactions controlled by a finely-tuned regulatory network [7]. Through the network, genes encoding protective proteins such as osmotin, mRNA binding proteins, and key enzymes for biosynthesis of metabolites are activated for osmotic adjustment [8]. The accumulation of metabolites like glycine betaine, proline, soluble sugars, and polyols can adjust osmotic homeostasis against chilling stress [9,10]. Furthermore, genes encoding stress responsive proteins, including various transcription factors, proteins kinases, and other signaling molecules, are also activated [11]. *C-REPEAT BINDING FACTOR* (*CBFs*)-controlled gene regulation mode is the most well-known mechanism associated with cold stress, but it only controls a share of the cold-responsive transcriptome. There are other *CBF*-independent regulatory mechanisms in plants. For example, *ZAT10* is a negative regulator of COR genes in which activity is repressed by the enolase *LOW EXPRESSION OF OSMOTICALLY RESPONSIVE GENES 2/C-MYC BINDING PROTEIN* (*LOS2/AtMBP-1*) under cold conditions [12]. 

Hormone changes also play an important role in response to chilling stress. Auxin (IAA) is required for plant root development. Exogenous IAA can promote root development [13]. A high level of IAA can maintain normal starch hydrolysis and sugar consumption to promote seed germination under low temperature [14]. Bio-active gibberellins (GAs) increase after seed imbibition, and this increase occurs just before radicle protrusion [15]. It has been reported that the reduction of GA accumulation along with the increase of abscisic acid (ABA) in seeds inhibits seed germination under chilling injury [16]. In addition, low temperature treatment decreases the content of cytokinin (CTK) and CTK oxidase in wheat [17]. Thus, better cold tolerance may be obtained by altering hormone levels. 

Seed germination is considered as the most critical developmental phase in the life cycle of seed plants. It requires coordination of complex cellular and biochemical processes, including cell cycle activation, hormonal balance, energy metabolism, and repair responses [18]. According to the water absorption rate, seed germination is divided into three phases. Phase I is characterized with rapid water absorption, and it is the initial stage of germination. At phase I, the cell membrane turns from gel phase to liquid crystal status, and the respiration of seeds is rapidly enhanced [19]. Phase II is named as the absorbing platform. It is the critical phase of germination because gene expression and metabolic activity are largely enhanced [20,21]. Phase III is indicated with the appearance of the hypocotyl and continued water absorption and seedling growth. It represents the post-germination period [22]. Normal seed germination and seedling development assure a good beginning of subsequent cultivation and harvest [23]. Therefore, it is important to understand the regulatory mechanisms of seed germination. 

Seed germination has been studied by using transcriptomic and proteomic analysis, either independently or in combination, in legumes [24,25], *Arabidopsis thaliana* [26], cereals [27], and other species [28]. In our study, two varieties of cotton exhibiting a significant difference in chilling response during germination were selected. The hypocotyl of KN27-3 (cold-tolerant variety) grew faster compared to XLZ38 (cold-susceptible variety) at 16 °C /4 °C. Their embryos from five hours post imbibition (hpi) of germination were harvested for transcriptome analysis and endogenous hormones test. The results characterized the major features of the chilling response during imbibition and identified low temperature related genes that can be used for molecular breeding.

## 2. Results

### 2.1. Morphological Characteristics of KN27-3 and XLZ38 during Germination under Chilling Stress

**Figure 1:**
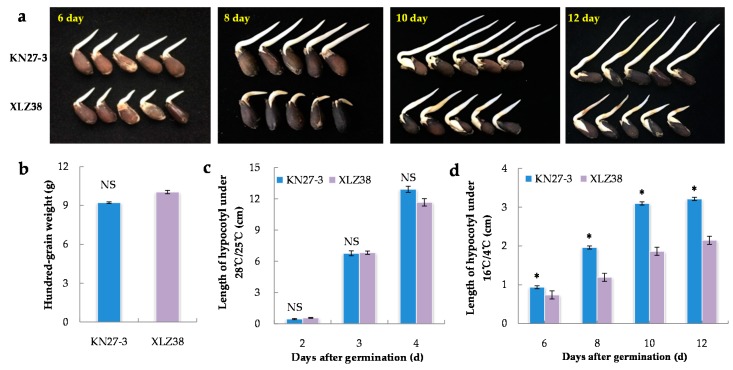
Morphological characteristics of KN27-3 and XLZ38 during germination under normal and chilling conditions. (**a**) Phenotypic differences between KN27-3 and XLZ38 on day 6, 8, 10, and 12 under 16 °C /4 °C; (**b**) hundred-grain weight of two varieties; length of hypocotyl after germination under 28 °C /25 °C (**c**) and 16 °C /4 °C (**d**). Bars marked with asterisks indicate differences change between the same hour (* *p* < 0.05) according to Duncan’s multiple range test using SPSS software. “NS” represents no difference.

Twenty-one different varieties of cotton were investigated for their hypocotyl length during germination under chilling conditions (Supplementary File S1). The biggest difference in seed germination speed was found between KN27-3 and XLZ38 (Figure 1a). Under normal conditions (28 °C/25 °C), there was no difference in either 100-grain weight (Figure 1b) or hypocotyl length (Figure 1c). Under chilling conditions (16 °C /4 °C), the hypocotyl length of KN27-3 was 0.94 cm, which was about 30% longer than that of XLZ38 on the 6^th^ day after imbibition. The length difference got larger on the 8^th^ day and 10^th^ day. On the 12^th^ day, the hypocotyl length of KN27-3 was about 45% longer than that of XLZ38 (Figure 1d), indicating that the KN27-3 variety was more cold-tolerant. 

### 2.2. RNA-Seq Analysis of the Two Varieties at the Early Germination Stage under Chilling Conditions and Identification of Differentially Expressed Genes (DEGs)

Under the 16 °C/4 °C conditions, cottonseeds began to absorb moisture at a faster rate for the first 42 h than 54 h, belonging to Phase I. From 42 h to 54 h, seeds barely absorbed water, belonging to Phase II (Supplementary Figure S1). In order to understand the reasons for the different chilling resistance in the two cotton varieties, samples at five imbibition time points were harvested for RNA extraction and RNA-Seq analysis. At each time point, three biological replicates were designed in both two varieties, and embryos at five imbibition time points were collected. A total of thirty samples were obtained.

**Figure 2:**
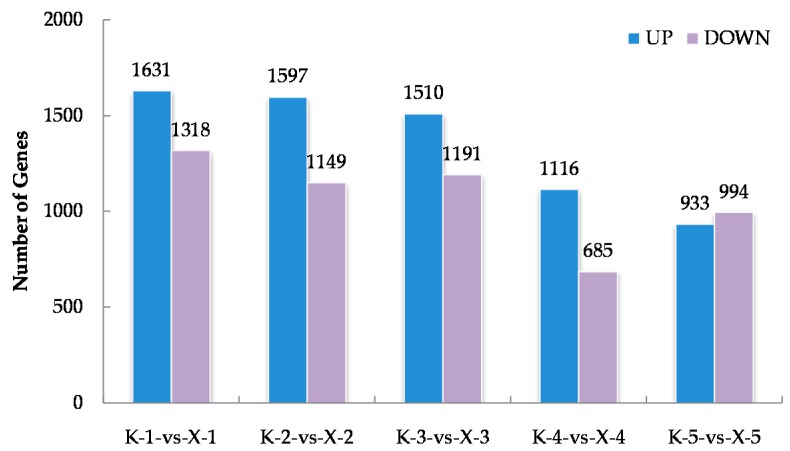
The statistics of up-regulated and down-regulated differentially expressed genes (DEGs) between KN27-3 and XLZ38 during germination under chilling conditions. K-1-vs-X-1, comparison between KN27-3 and XLZ38 at stage 1. K-2-vs-X-2, comparison between KN27-3 and XLZ38 at stage 2; K-3-vs-X-3, comparison between KN27-3 and XLZ38 at stage 3; K-4-vs-X-4, comparison between KN27-3 and XLZ38 at stage 4; K-5-vs-X-5, comparison between KN27-3 and XLZ38 at stage 5. Stages 1-5 represent the sampling hours post imbibition at 12, 18, 30, 42, and 54 h.

Approximately 1.41 × 10^9^ raw reads were generated for the thirty samples via RNA-Seq whereas, 98.28% clean reads were obtained (Supplementary File S2). By anchoring the clean reads to the cotton reference genome, we obtained the expression data of 76,331 genes (Supplementary File S3). The *Arabidopsis thaliana* database was then used to determine the functional annotations of cotton genes using the BLAST algorithm. To further identify the genes closely correlated with the phenotypes of the two varieties, differentially expressed genes (DEGs) were further obtained by comparing their expression levels in KN27-3 and XLZ38 at five imbibition stages. The results show 1631, 1597, 1510, 1161, and 933 genes significantly up-regulated XLZ38 relative to KN27-3 at stages one to five, while 1318, 1149, 1191, 685, and 994 genes were down-regulated. There were a total of 7535 genes expressed differentially (Figure 2). There were more up-regulated genes than down-regulated ones from stage one to stage four.

### 2.3. KEGG Pathway Analysis of DEGs 

**Figure 3:**
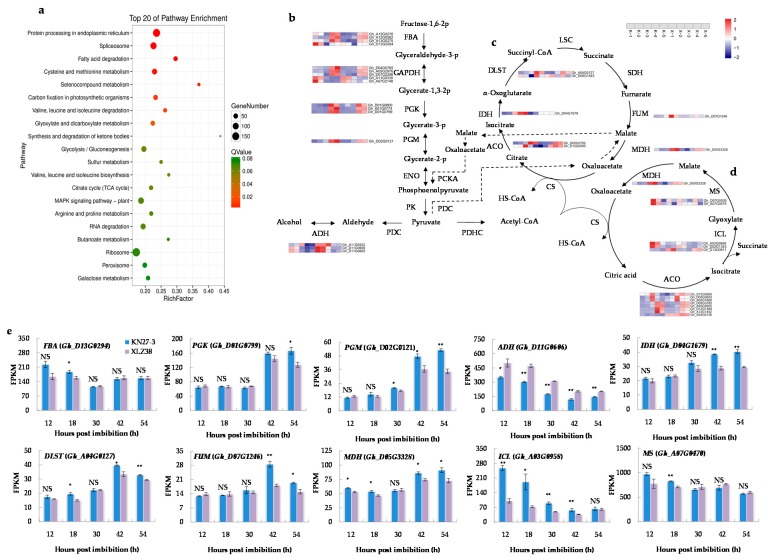
Kyoto Encyclopedia of Genes and Genomes (KEGG) pathway enrichment analysis of DEGs. (**a**) The top 20 enriched pathways. The colors are shaded according to the Q-values level, as shown in the color bars gradually from low (red) to high (green); the size of the circle indicates the number of DEGs from small (less) to big (more); (**b**) glycolysis/gluconeogenesis pathways connected by black straight arrow line/black dashed arrow line and tricarboxylic acid (TCA) cycle (**c**) and glyoxylate cycle (GAC) metabolism (**d**) shown by the loop diagram. Relative levels of DEG expression are shown by a color gradient from low (blue) to high (red); (**e**) fragments per kilobase of transcripts per million mapped fragments (FPKM) of different major genes involved in the above metabolic pathways at different hours post imbibition. K, KN27-3. X, XLZ38. The numbers along the X-axis represent the sampling hours post imbibitions. The numbers in the scale bars indicate the log2 (fold changes) in gene expression. Bars marked with asterisks indicate differences change between the same hour (* *p* < 0.05, ** *p* < 0.01) according to Duncan’s multiple range test using SPSS software. “NS” represents no difference.

To further elucidate the functions of the DEGs in response to chilling stress, Kyoto Encyclopedia of Genes and Genomes (KEGG) databases were used to classify DEGs into the corresponding pathways. Twenty KEGG pathways were significantly enriched in five comparisons (Figure 3a, Supplementary File S4). Carbon metabolism was an essential process in plants to produce both energy sources and structural components of cells. In our study, carbon metabolism was the most enriched pathway containing 194 DEGs involved in “glyoxylate and dicarboxylate metabolism” (47 unigenes), “glycolysis/gluconeogenesis” (71 unigenes), “citrate cycle” (38 unigenes), and “galactose metabolism” (38 unigenes). As shown in Figure 3b, the expression changes of most DEGs involved in glycolysis/gluconeogenesis exhibited the same dynamic trend in both varieties, but the increase was significantly stronger in KN27-3 at 42 and 54 h than in XLZ38 (Figure 3b, Supplementary File S5). These genes encode fructose-bisphosphate aldolase (FBA), phosphoglycerate kinase (PGK), and phosphoglycerate mutase (PGM). In addition, the genes encoding alcohol dehydrogenase (ADH) with a function in ethanol production under anaerobic conditions was significantly more up-regulated at 12, 18, and 30 hpi in XLZ38.

Pyruvate (PEP) produced by glycolysis entered the tricarboxylic acid cycle (TCA cycle) and glyoxylate cycle (GAC) under aerobic conditions (Supplementary File S6). The DEGs involved in the TCA cycle (Figure 3c) were significantly more up-regulated from 42 to 54 hpi in KN27-3, such as *isocitrate dehydrogenase* (*IDH*), *dihydrolipoamide succinyltransferase* (*DLST*), *fumarase* (*FUM*), and *malate dehydrogenase* (*MDH*). The DEGs involved in GAC (Figure 3d), *isocitrate lyase* (*ICL*) and *malate synthase* (*MS*), which encode unique enzymes for GAC, were significantly up-regulated in KN27-3 at 12 and 18 hpi, while the expression of *MDH* kept increasing from 42 to 54 hpi. In summary, energy came from the GAC metabolism in KN27-3 at the early stage of imbibition (from 12 to 18 hpi), while it might come from an aerobic respiration in XLZ38. At the later stage of germination (from 42 to 54 hpi), the energy came from glycolysis and the TCA cycle for hypocotyl elongation in KN27-3. 

### 2.4. GO Function Enrichment Analyses of DEGs

**Figure 4:**
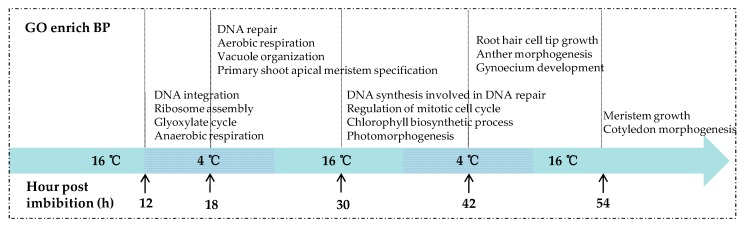
Gene Ontology (GO) categories of up-regulated DEGs were significantly enriched in the biological processes (BP) category at 12, 18, 30, 42, and 54 h post imbibition. The x-axis shows culture temperatures and hours.

DEGs obtained from the five comparisons were used to search against the Gene Ontology (GO) database and the analysis of GO categories was performed. The results showed that the biological processes (BP) related to seed germination were significantly enriched earlier in KN27-3, indicating that KN27-3 developed faster than XLZ38. Figure 4 shows GO terms with up-regulated genes enrichment in KN27-3, which were not present in the down-regulated genes enrichment results (Supplementary File S7). After the seeds absorbed water for 12 hours, DNA integration and RNA assembly were enriched. Anaerobic respiration and glyoxylate metabolism were enriched in the embryo. At 18 hpi, DNA was repaired, vacuole formed, the seed switched from anaerobic respiration to aerobic, meristematic cells started differentiation to form new organs, DNA began to replicate, and mitosis was activated at 30 hpi. In addition, the synthesis of chlorophyll and photomorphogenesis were also significantly enriched. At 42 h, hypocotyls were developing, anther and gynoecium development emerged in KN27-3. At the last 54 hpi, meristem continued to grow and the cotyledon began to form in KN27-3 (Figure 4). XLZ38 had a similar growth pattern throughout but was delayed compared to KN27-3. 

### 2.5. DEGs Related to Osmoregulation

**Figure 5:**
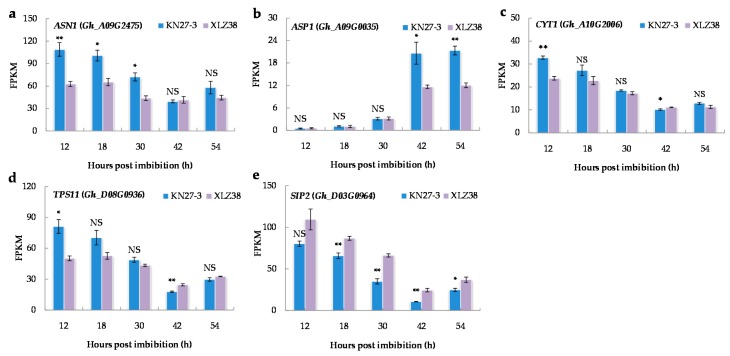
Major DEGs related to osmoregulation. (**a**) *ASN1*, (**b**) *ASP1*, (**c**) *CYT1*, (**d**) *TPS11*, (**e**) *SIP2*. Values are the FPKM means of three independent replicates ± SE. Bars marked with asterisks indicate differences change between the same hour (* *p* < 0.05, ** *p* < 0.01) according to Duncan’s multiple range test using SPSS software. “NS” represents no difference.

After the onset of chilling, plants often increase the accumulation of osmotic-related substances to reduce water loss. Therefore, the osmoregulation associated genes were analyzed. According to the GO enrichment analysis, asparagine biosynthesis occurred in KN27-3 at all five stages analyzed (Supplementary File S8), but it occurred in XLZ38 only at 30 hpi. Glutamine-dependent asparagine synthetase 1 (ASN1) is an important enzyme in the nitrogen metabolism cycle of plants and a key enzyme for asparagines synthesis. *ASN1* (*Gh_A09G2475*) was much more expressed from 12 to 30 h in KN27-3 than in XLZ38 but showed a slight decrease afterward while maintaining a low level in XLZ38 (Figure 5a). ASP1 catalyzes transamination between aspartate and oxaloacetate, the expression of *aspartate aminotransferase 1* (*ASP1*) (*Gh_A09G0035*) showed a notable increase in both KN27-3 and XLZ38 at 42 hpi and remained at similar levels at 54 h, but its fragments per kilobase of transcripts per million mapped fragments (FPKM) in KN27-3 was nearly twice that in XLZ38 at 42 and 54 h (Figure 5b). 

Seed germination was accompanied by soluble sugar synthesis and catabolism. GDP-mannose biosynthetic process (GO: 0009298) and trehalose biosynthetic process (GO: 0005992) were enriched in KN27-3 at 12 hpi accompanied by the high up-regulation of the genes involved (Supplementary File S9). *CYT1* encoded a GDP-mannose pyrophosphorylase/mannose-1-pyrophosphatase and *TPS11* encoded an enzyme putatively involved in trehalose biosynthesis. *CYT1* (*Gh_A10G2006*) and *TPS11* (*Gh_D08G0936*) had higher expression levels at 12 hpi in KN27-3, and then lowered with the prolonged germination time of two varieties (Figure 5c,d). Raffinose catabolic process (GO: 0034484) was found to be active in KN27-3 at all analyzed stages except stage 4. The enriched genes were also up-regulated in XLZ38 (Supplementary File S9). *SIP2* (*Gh_D03G0964*) encodes a raffinose-specific alpha-galactosidase that catalyzes the breakdown of raffinose into alpha-galactose and sucrose. Its expression level in XLZ38 was higher than in KN27-3 during the entire germination process (Figure 5e). The expression of both anabolic and catabolism-related genes showed higher expression levels from 12 to 30 hpi. Thus, the early up-regulation of the genes involved in the biosynthesis of mannose, trehalose, and raffinose in KN27-3 may provide useful protection against chilling stress. 

### 2.6. DEGs Involved in Synthesis of Antioxidants and Antioxidative Enzymes

**Figure 6:**
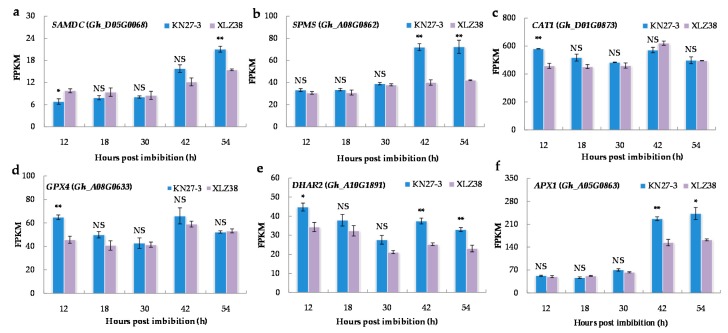
The expression of DEGs related to spermine and polyamine biosynthetic process and antioxidant activity. (**a**) *SAMDC*, (**b**) *SPMS*, (**c**) *CAT1*, (**d**) *GPX4*, (**e**) *DHAR2*, (**f**) *APX1*. The Y axis shows the FPKM means of three independent replicates ± SE. Bars marked with asterisks indicate differences change between the same hour (* *p* < 0.05, ** *p* < 0.01) according to Duncan’s multiple range test using SPSS software. “NS” represents no difference.

Antioxidants play an important role in plant resistance to low temperatures. Therefore, the expression of the genes involved in antioxidant biosynthesis was analyzed. The major genes are shown in Figure 6 and other related genes in Supplementary File S10. The spermine biosynthetic process (GO: 0006597) and polyamine biosynthetic process (GO: 0006595) were found to be significantly enriched in KN27-3 at 54 hpi. For example, S-adenosylmethionine decarboxylase (SAMDC) is a key enzyme in the polyamine metabolic pathway. Its expression was remarkably more up-regulated at 54 h in KN27-3 compared to XLZ38 (Figure 6a). In addition, *Gh_A08G0862* encoding spermine synthase (SPMS) was expressed at a higher level in KN27-3 at 42 and 54 hpi compared to that in XLZ38, which remained unchanged (Figure 6b).

Catalase isozyme (CAT), glutathione peroxidase (GPX), glutathione S-transferase (DHAR), and L-ascorbate peroxidase (APX) were activated during germination. The related DEGs, including *Gh_D01G0873*, *Gh_A08G0633*, and *Gh_A10G1891,* are listed in Supplementary File S11. *CAT1*, *GPX4*, and *DHAR2* were up-regulated with high expression in KN27-3 at 12 hpi (Figure 6c–e). *APX1* (*Gh_A05G0863*) had a superior FPKM value from 42 to 54 h (Figure 6f). In summary, the genes encoding enzymes for antioxidant synthesis and antioxidant activity showed similar trends in the two varieties with higher expression in KN27-3. 

### 2.7. Endogenous Hormone Content and Related Gene Expression

**Figure 7:**
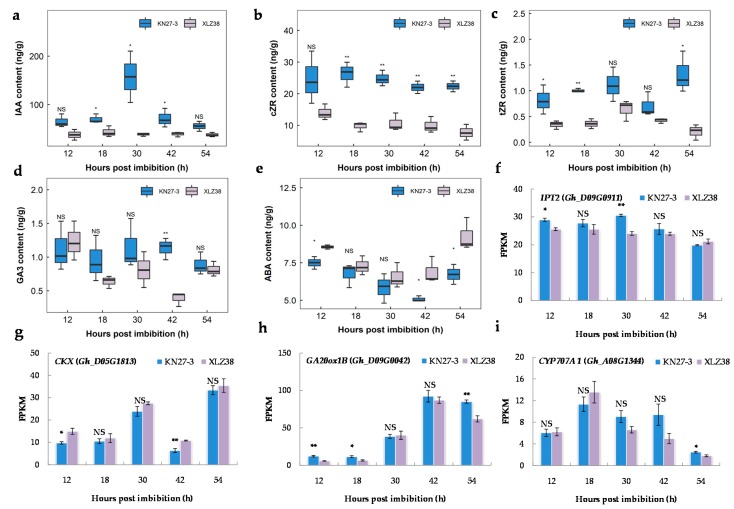
Endogenous hormone contents in KN27-3 and XLZ38 and the expression of the genes related to hormone metabolism during cottonseed germination. (**a**) IAA, (**b**) cZR, (**c**) tZR, (**d**) GA3, (**e**) ABA, (**f**) *IPT2*, (**g**) *CKX*, (**h**) *GA20ox1B*, (**i**) *CYP707A1*. Y-axis values are the means of three independent replicates ± SE. Bars marked with asterisks indicate differences change between the same hour (* *p* < 0.05, ** *p* < 0.01) according to Duncan’s multiple range test using SPSS software. “NS” represents no difference.

Plant hormones are important for seed development and response to chilling stress; therefore, the contents of endogenous hormones at five germination stages were measured via high performance liquid chromatography-mass spectrometry (HPLC-MS). As shown in Figure 7a, higher IAA content was detected in KN27-3 at all time points with a maximum level of 157.40 ng/g at 30 hpi, which was about four-fold higher than that of XLZ38. There was also more accumulation of cZR and tZR in KN27-3 compared to XLZ38 (Figure 7b,c). The GA3 content (Figure 7d) in the embryos of the two varieties showed a large difference at 42 hpi, which was about three times higher in KN27-3 than that of XLZ38. While ABA content in XLZ38 was higher than in KN27-3 at all five germination stages with a significant difference at 12, 42, and 54 hpi (Figure 7e). 

DEGs involved in CKT, GA, and ABA metabolism were identified, and their changes during seed germination were consistent with the accumulation of hormones. TP/ADP isopentenyl transferase 2 (IPT2) catalyzes the rate-limiting step of CTK biosynthesis. Its encoding gene *Gh_D09G0911* had a higher FPKM value in KN27-3 than in XLZ38. Its expression reached its peak at 30 hpi and declined later (Figure 7f). Moreover, *Gh_D05G1813*, encoding oxidase/dehydrogenase, which is involved in CTK degradation, was up-regulated in XLZ38 and was significantly higher than in KN27-3 at 12 and 42 hpi (Figure 7g). *Gh_D09G0042* encoding GA20 oxidase homolog GA20ox1B was expressed during germination. It is involved in GA biosynthesis. *GA20ox1B* had significantly higher expression in KN27-3 than that in XLZ38 at 12 and 18 hpi. In KN27-3, its expression was stable but significantly down-regulated in XLZ38 at 54 h compared to 42 h (Figure 7h). Abscisic acid 8’-hydroxylase 1 (CYP707A1) is an important enzyme for ABA inactivation. *Gh_A08G1344* was down-regulated in XLZ38 from 30 h to 42 h, while it was not changed in KN27-3. Its expression first increased and then decreased by 18 h in both varieties, although the decrease was faster in XLZ38 than in KN27-3 (Figure 7i). In summary, the expression dynamics for these genes was consistent with the hormone accumulative dynamics in the two varieties. IAA, GA3, and CTK played a positive regulatory role, and ABA played a negative regulatory role in KN27-3 during the low temperature germination.

## 3. Discussion

Plants growing in the natural environment are constantly subjected to a variety of abiotic stresses, such as drought, salinity, heat, cold, and heavy metals. Plant stress response and adaptation are extremely complex, involving molecular, cellular and physiological changes [29]. Based on the present study, seed germination rates of varieties, KN27-3 and XLZ38 showed a significant difference at low temperatures, indicating different responses to chilling (Supplementary File S1). Our results demonstrated that early and rapid response to low temperature in KN27-3 played an important role in promoting its hypocotyl elongation. KN27-3 ensured the stability of the intracellular environment to reduce chilling damages. It is known that enhancing the biosynthesis of different types of compatible organic solutes is one of the most common responses of plants against stress [30]. Asparagines as the main form of transport of amino acids in cotton was not only an important carrier for transporting new synthetic nitrogen in plants but also a potential osmotic adjustment [31,32]. Stewart found that asparagine might have a function similar to proline-regulating cell penetration [31,33]. *ASP1* and *ASN1* had high expression levels during the germination of KN27-3. ASP1 and ASN1 catalyze the production of aspartate and asparagine, respectively. In our results, more aspartic acid and asparagine were produced in the seed germination of KN27-3 to regulate the osmotic pressure of cells, which may protect the germination process from the chilling conditions in addition to producing more ammonium.

The biosynthesis process of trehalose and GDP-mannose were enriched during the germination of the two varieties. *CYT1* encoding GDP-mannose pyrophosphorylase/mannose-1-pyrophosphatase had a higher expression level in KN27-3 at 18 h. Over-expression of tomato GMPase in tobacco reduced H_2_O_2_ and O^2−^ content in plants under high temperature or low temperature stress [34]. GMPase was significantly up-regulated in KN27-3, which may reduce the stress-induced peroxidative damage and produce the structural component, GDP-mannose, for the cell wall. The synthesis of carbohydrates was also the substrate for the synthesis of trehalose [35]. Chickpea with accumulative trehalose performed better under both optimal temperature conditions and chilling stress because trehalose protects the plants from oxidative damage and helps to maintain carbon assimilation and seedling growth [36]. In the present study, four *TPS11/9* homologous genes had higher expression levels from 12 to 30 h in KN27-3 similar to *ASN1* and *CYT1*. The *TaTPS11* transcription level was positively correlated with wheat cold tolerance [37]. The expression of *AtTPS6*, *7*, *9,* and *10* persists towards the root base and stops at the root/hypocotyl junction, promoting hypocotyl elongation in *Arabidopsis* [38]. Cold stress-induced raffinose synthesis can regulate plant permeability for cold resistance [39]. Raffinose family oligosaccharides (RFO) facilitate chickpea seed germination [40]. The catabolism of raffinose was enriched to almost the entire imbibition process. The degree of raffinose decomposition in XLZ38 was higher than that of KN27-3, which may lead to more raffinose in KN27-3 and improve stress tolerance (Figure 5e). Therefore, during the imbibition stage, more GDP-mannose and trehalose may be synthesized, while more raffinose may be broken down in the early germination stage (from 12 to 30 hpi) of KN27-3, which helps with osmotic adaptation under low temperatures during seed germination.

The embryo development is often associated with the accumulation of reactive oxygen species (ROS) [41]. ROS is also generated in tissues under chilling stress. And more ROS accumulation can cause different degrees of damage to proteins, lipids, nucleic acids, and other biological macromolecules, which may lead to the change of biofilm fluidity and inactivation of biological enzymes, hinder protein synthesis and degradation, and cause DNA damage, etc. [42]. Therefore, an effective antioxidant mechanism is regarded as a prerequisite for obtaining vigorous seed. The damages caused by chilling stress are correlated with unbalanced intracellular oxidative systems in plants [43]. All polyamines have the ability to bind DNA for protection. They also contribute to plant stress tolerance by minimizing oxidative damage and maintaining membrane structure [44]. Spermidine (Spd) was shown to enhance clover seed germination under low temperature [45]. In our study, DEGs involved in SAMDC and SPMS were expressed in both cultivars, but higher expression levels were observed in KN27-3 than in XLZ38 during seed germination. Multiple studies have demonstrated that Spd is generally correlated with the hypocotyl elongation rate, such as in chickpea [46], corn [47], and in mung bean [48]. Thus, higher expression of SAMDC and SPMS may increase the accumulation of Spd in germinating KN27-3 seeds under chilling conditions. 

Stress, for example, water shortage [49] and K deficiency [50], can increase the activities of antioxidant enzymes for scavenging ROS, while low temperatures may inhibit enzyme activity [51]. Thus, it is likely that the fine-tuned regulation of antioxidant enzyme activities was involved in promoting hypocotyl elongation under chilling conditions in KN27-3. The expression of *CAT1*, *GPX4*, *DHAR2*, and *APX1* in KN27-3 was higher than that in XLZ38 (Figure 6). Rapid and efficient expression of *CAT1* and *GPX4* was more pronounced at 12 hpi, while *APX1* was more strongly expressed at later time points (42 and 54 h), and *DHAR2* was expressed throughout the germination stage (12, 42, and 54 h). GPX4 is present in various cell compartments and it closely cooperates with enzymes, such as SOD and CAT1, to coordinate and eliminate excessive free radicals in plants and improve plant resistance [52]. Under drought and cold stress conditions, glutathione (GSH) and APX are responsible for clearing drought and cold-induced ROS detoxification, and their increased expression promotes higher germination rates, increased root length and higher accumulation of fresh weight [53]. Studies have shown that higher CAT activity can reduce H_2_O_2_ content in plants [54] and that elevated CAT activity can improve rice tolerance and seed germination rate at low temperatures [55]. Our data suggest that KN27-3 has a higher ability to alleviate the harmful effects of ROS under cold stress than XLZ38, confirming that rapid accumulation of antioxidants is an important factor in determining cottonseed cold stress tolerance.

Various studies have shown that plant hormones regulate seed germination. ABA and GAs play the most important roles, while IAA is present in the seedling radicle tip during and after germination, and CTK is activated during germination [56,57]. Our data suggest that IAA and CTK accumulated during germination of KN27-3 under chilling stress, which serves to ensure the growth of embryos (Figure 7a–c). When plants are stressed, they maintain growth by balancing both IAA and CTK hormonal pathways along with ROS signals [58]. IPT2 catalyzes the decomposition of prenylated tRNA, which is a key step in cZT synthesis. The occurrence of zeatin cis-trans isomerase activity suggested that the tRNA-mediated pathway might also contribute to the synthesis of tZ-type CKs via cZ-type CKs [59]. CKX enzymes catalyze their reversible degradation of CTK by oxidative side chain cleavage [58]. Our results showed that the transcripts *Gh_D09G0911*, encoding IPT2, and *Gh_D05G1813*, encoding CKX, were up-regulated and down-regulated, respectively, to produce more CTK in KN27-3. During the germination of dicotyledons, GA mainly stimulates germination by promoting radicle elongation and penetration of the seed coat [60]. Our data suggest that the accumulation of GA3 in KN27-3 began to increase at 30 h, and was significantly higher than that of XLZ38 at 42 h. Expression of the gene encoding GA20ox1B also significantly increased in KN27-3 at 42 h, and there was a significant difference at 54 hpi (Figure 7d,h). This suggests that the synthesis of GA3 positively regulated the germination process of KN27-3 under chilling stress. ABA is a key phytohormone that modulates plant growth and development as well as abiotic and biotic stress responses. ABA accumulates in the developing embryo and regulates seed development, seed maturation, and seed dormancy [61]. ABA endogenous content was higher in XLZ38 (Figure 7e). *CYP707A1* is one of the two important ABA catabolic genes [62]. *Gh_A08G1344* transcription levels were down-regulated during germination in XLZ38 (Figure 7i). We speculated that ABA inhibits seed germination while IAA, CTK, and GA3 positively regulates seed germination, suggesting that hormones and transcription of metabolic genes together regulate the germination process of seeds.

Antioxidase and catalytic enzymes involved in the metabolism of the two varieties have different expression levels of transcription under the same temperature. In the next research, we will continue to study the reasons for the different antioxidant enzyme activity of different varieties at the same temperature, and explore the relationship between the level of antioxidant enzyme activity and the content of H_2_O_2_ and O^2−^ content in cottonseed under chilling conditions.

## 4. Materials and Methods 

### 4.1. Plant Material and Seed Germination Conditions

Two varieties were used in the experiments: a cold-susceptible variety XinLuZao38 (XLZ38) and a cold-tolerant material KenN27-3 (KN27-3). Both varieties are suitable for planting in the northwest inland cotton region (Xinjiang, China). Their seeds were harvested in Xinjiang in 2017.

The experiments were performed in a germination box (185 × 145 × 62 mm) with 12 holes. Plants were cultured in sand with a water content of 13%. Five seeds were placed in each hole, a total of 20 seeds per variety were planted. Three replicates were performed. 

Cotton seeds were grown (28 °C/25 °C, 12 h/12 h, day/night) for 4 days to analyze the seed germination phenotype. The hypocotyls lengths were measured at 2, 3, and 4 days after sowing. Germination tests under chilling stress were performed in a growth chamber with ~150 μmol m^−2^s^−1^ of fluorescent light, a 12 h day/12 h night cycle, and 16 °C/4 °C, and hypocotyl length was determined 6, 8, 10, and 12 days after the initiation. Fresh seed weight was measured the weight every 6 h, and water absorption per unit mass of cotton seed (ΔM) was calculated as ΔM = (M_i_ − M_0_)/M_0_, in which M_0_ is the dry weight of 20 seeds, M_i_ is the weight of 20 seeds at different germination times. Seed embryos of KN27-3 and XLZ38 (named K1-5, X1-5) were collected at five germination stages: 12, 18, 30, 42, and 54 h (Supplemental Figure S2), frozen in liquid nitrogen and stored at −80 °C for RNA-Seq and hormone determination.

### 4.2. RNA Isolation, Library Construction, and RNA-Seq 

After total RNA was extracted, eukaryotic mRNA was enriched by Oligo (dT) beads, while prokaryotic mRNA was enriched by removing rRNA by Ribo-Zero^TM^ Magnetic Kit (Epicentre). Then the enriched mRNA was fragmented into short fragments using fragmentation buffer and reverse transcribed into cDNA with random primers. Second-strand cDNA was synthesized by DNA polymerase I, RNase H, dNTP, and buffer. Then, the cDNA fragments were purified with a QIAquick PCR extraction kit, end repaired, poly(A) added, and ligated to Illumina sequencing adapters. The ligation products were size selected by agarose gel electrophoresis, PCR amplified, and sequenced using Illumina HiSeq^TM^ 2500 by Gene Denovo Biotechnology Co. (Guangzhou, China).

### 4.3. Sequencing Analysis and Differential Expression Analysis

Clean reads were obtained by removing adaptor sequences, more than 10% N bases and low-quality (Q ≤ 20) reads with more than 50% bases from each data set to gain more reliable results. The reads were mapped onto the transcriptome assembly by TopHat 2 (V2.1.1) to map the reads to the *Gossypium hirsutum* L. genome [63]. Read counts per gene were expressed as the expected number of fragments per kilobase of transcripts per million mapped fragments (FPKM), and unigene abundance differences between the samples were calculated based on the ratio of the FPKM values and false discovery rate (FDR). Genes with FDR  ≤  0.05 and FPKM ≥ 10 were considered DEGs. 

GO classification was performed via WEGO, (http://wego.genomics.org.cn/cgi-bin/wego/index.pl), and the GO distributions of DEGs were then obtained from three levels: biological process and cellular component, molecular function. For each Kyoto Encyclopedia of Genes and Genomes (KEGG) pathway, the numbers of DEGs were compared to the entire reference gene set by hypergeometric tests to find out the pathways enriched with regulated genes. KEGG enrichment analyses were adjusted using the Bonferroni correction, and a corrected *p*. adjust ≤ 0.05 was chosen as the threshold value for determining significantly enriched GO terms and KEGG enrichment pathways. 

### 4.4. Measurements of Various Hormones

Endogenous hormone content was measured for samples of KN27-3 and XLZ38. Each sample was prepared in triplicate. Endogenous indoleacetic acid (IAA), cytokinins (CKT), including cis-zeatin riboside (cZR) and trans-zeatin riboside (tZR), gibberellins (GA), and abscisic acid (ABA) contents were determined using an ultra-performance liquid chromatography-electrospray ionization-tandem mass spectrometry (UPLC-ESI-MS/MS) system [64].

### 4.5. Statistical Analysis 

The statistical analysis was conducted with SPSS software 22 (SPSS. Inc) for Windows. One-Way ANOVA analysis and Tukey’s honestly significant difference test were performed to test for differences between the means of the embryo data for KN27-3 and XLZ38 at different stages using a significance level of *p* < 0.05.

## 5. Conclusions

In total, 7535 DEGs were identified and analyzed for their potential role between KN27-3 and XLZ38 at five imbibition stages using GO enrichment and KEGG pathway analysis. Elevated levels of IAA, CTK, and GA and reduced ABA in KN27-3 (tolerant genotype) might interact with one another to alter energy metabolism to maintain nitrogen and carbon balance, which might contribute to seed germination. Ultimately, asparagine, GDP-mannose, trehalose, and raffinose metabolic process, the rapid accumulation of spermine, and higher antioxidase activity of genotype KN27-3, may explain its improved performance under chilling stress.

## Supplementary Materials

Supplementary materials can be found at https://www.mdpi.com/1422-0067/21/6/2067/s1. Supplementary File S1. Preliminary experiments about twenty-one varieties; Supplementary File S2. Detailed information on the obtained reads via RNA-Seq; Supplementary File S3. Expression of all genes in the 30 samples; Supplementary File S4. KEGG pathway enrichment analysis of DEGs; Supplementary File S5. List of DEGs related to glycolysis/gluconeogenesis; Supplementary File S6. List of DEGs related to the TCA cycle and GAC; Supplementary File S7. GO terms were all high-profile enrichment in KN27-3; Supplementary File S8. Asparagine biosynthetic process about amino acids enriched in GO term; Supplementary File S9. Trehalose, GDP-mannose, and raffinose metabolism process enriched in GO term; Supplementary File S10. List of DEGs related to polyamine metabolic process; Supplementary File S11. List of genes related to antioxidase; Supplementary Figure S1. Cottonseed water content changes during seed germination of KN27-3 and XLZ38 at 16 °C/4 °C; Supplementary Figure S2. Schematic diagram of germination process and sampling time.

## References

[1] Pham H.M., Kebede H., Ritchie G., Trolinder N., Wright R.J. (2018). Alternative oxidase (AOX) over-expression improves cell expansion and elongation in cotton seedling exposed to cool temperatures. Theor. Appl. Genet..

[2] Lu X., Jia X., Niu J. (2018). The present situation and prospects of cotton industry development in China. Sci. Agric. Sin..

[3] Bartkowski E.J., Buxton D., Katterman R., Kircher H.W. (1977). Dry seed fatty acid composition and seedling emergence of pima cotton at low soil temperatures. Agron. J..

[4] Yu Y.W., Yu G.X., Wei J.Z. (2019). Analysis of spatial distribution and influencing factors of cotton production lay-out in Xinjiang under the backdrop of supply side reform. J. Arid Land Resour. Environ..

[5] Ashraf M. (2002). Salt tolerance of cotton: Some new advances. Crit. Rev. Plant Sci..

[6] Yan L. (2013). Responses to cold stress of six cotton varieties in initial growth stage. Acta Laser Biol. Sin..

[7] Raju S.K.K., Barnes A.C., Schnable J.C., Roston R.L. (2018). Low-temperature tolerance in land plants: Are transcript and membrane responses conserved?. Plant Sci..

[8] Yamaguchi-Shinozaki K., Shinozaki K. (2006). Transcriptional regulatory networks in cellular responses and tolerance to dehydration and cold stresses. Annu. Rev. Plant Biol..

[9] Jorge T.F., Rodrigues J.A., Caldana C., Schmidt R., Van Dongen J.T., Thomas-Oates J., António C. (2016). Mass spectrometry-based plant metabolomics: Metabolite responses to abiotic stress. Mass Spectrom. Rev..

[10] Kishitani S., Watanabe K., Yasuda S., Arakawa K., Takabe T. (1994). Accumulation of glycine betaine during cold acclimation and freezing tolerance in leaves of winter and spring barley plants. Plant Cell Environ..

[11] Seki M., Kamei A., Yamaguchi-Shinozaki K., Shinozaki K. (2003). Molecular responses to drought, salinity and frost: Common and different paths for plant protection. Curr. Opin. Biotechnol..

[12] Fowler S., Thomashow M.F. (2002). Arabidopsis transcriptome profiling indicates that multiple regulatory pathways are activated during cold acclimation in addition to the CBF cold response pathway. Plant Cell.

[13] Laskowski M., Biller S., Stanley K., Kajstura T., Prusty R. (2006). Expression profiling of auxin-treated *Arabidopsis* roots: Toward a molecular analysis of lateral root emergence. Plant Cell Physiol..

[14] Du H., Liu H., Xiong L. (2013). Endogenous auxin and jasmonic acid levels are differentially modulated by abiotic stresses in rice. Front. Plant Sci..

[15] Wang Y., Cui Y., Hu G., Wang X., Chen H., Shi Q., Xiang J., Zhang Y., Zhu D., Zhang Y. (2018). Reduced bioactive gibberellin content in rice seeds under low temperature leads to decreased sugar consumption and low seed germination rates. Plant Physiol. Biochem..

[16] Jiang L., Liu S., Hou M., Tang J., Chen L., Zhai H., Wan J. (2006). Analysis of QTLs for seed low temperature germinability and anoxia germinability in rice (*Oryza sativa* L.). Field Crop Res..

[17] Yuldashev R., Avalbaev A., Bezrukova M., Vysotskaya L., Khripach V., Shakirova F. (2012). Cytokinin oxidase is involved in the regulation of cytokinin content by 24-epibrassinolide in wheat seedlings. Plant Physiol. Biochem..

[18] Nonogaki H., Bassel G.W., Bewley J.D. (2010). Germination—Still a mystery. Plant Sci..

[19] Crowe J., Crowe L. (1992). Membrane integrity in anhydrobiotic organisms: Toward a mechanism for stabilizing dry cells. Water Life.

[20] Han C., Zhen S., Zhu G., Bian Y., Yan Y. (2017). Comparative metabolome analysis of wheat embryo and endosperm reveals the dynamic changes of metabolites during seed germination. Plant Physiol. Biochem..

[21] Howell K.A., Narsai R., Carroll A., Ivanova A., Lohse M., Usadel B., Millar A.H., Whelan J. (2009). Mapping metabolic and transcript temporal switches during germination in rice highlights specific transcription factors and the role of RNA instability in the germination process. Plant Physiol..

[22] Finch-Savage W.E., Leubner-Metzger G. (2006). Seed dormancy and the control of germination. New Phytol..

[23] Rosental L., Nonogaki H., Fait A. (2014). Activation and regulation of primary metabolism during seed germination. Seed Sci. Res..

[24] Gallardo K., Le Signor C., Vandekerckhove J., Thompson R.D., Burstin J. (2003). Proteomics of Medicago truncatula seed development establishes the time frame of diverse metabolic processes related to reserve accumulation. Plant Physiol..

[25] Hajduch M., Ganapathy A., Stein J.W., Thelen J.J. (2005). A systematic proteomic study of seed filling in soybean. Establishment of high-resolution two-dimensional reference maps, expression profiles, and an interactive proteome database. Plant Physiol..

[26] Ruuska S.A., Girke T., Benning C., Ohlrogge J.B. (2002). Contrapuntal networks of gene expression during Arabidopsis seed filling. Plant Cell.

[27] Grimanelli D., Perotti E., Ramirez J., Leblanc O. (2005). Timing of the maternal-to-zygotic transition during early seed development in maize. Plant Cell.

[28] Winkelmann T., Heintz D., Van Dorsselaer A., Serek M., Braun H.P. (2006). Proteomic analyses of somatic and zygotic embryos of *Cyclamen persicum* Mill. reveal new insights into seed and germination physiology. Planta.

[29] Rodziewicz P., Swarcewicz B., Chmielewska K., Wojakowska A., Stobiecki M. (2014). Influence of abiotic stresses on plant proteome and metabolome changes. Acta Physiol. Plant.

[30] Esmaeilpour A., Van Labeke M.C., Samson R., Van Damme P. (2015). Osmotic stress affects physiological responses and growth characteristics of three pistachio cultivars. Acta Physiol. Plant..

[31] Lea P.J., Sodek L., Parry M.A., Shewry P.R., Halford N.G. (2007). Asparagine in plants. Ann. Appl. Biol..

[32] Herrera-Rodríguez M.B., Pérez-Vicente R., Maldonado J.M. (2007). Expression of asparagine synthetase genes in sunflower (*Helianthus annuus*) under various environmental stresses. Plant. Physiol. Biochem..

[33] Stewart G., Larher F. (1980). Accumulation of amino acids and related compounds in relation to environmental stress. Amino Acids Deriv..

[34] Wang H.S., Yu C., Zhu Z.J., Yu X.C. (2011). Overexpression in tobacco of a tomato GMPase gene improves tolerance to both low and high temperature stress by enhancing antioxidation capacity. Plant. Cell Rep..

[35] Zakrzewska A., Palamarczyk G., Krotkiewski H., Zdebska E., Saloheimo M., Penttilä M., Kruszewska J.S. (2003). Overexpression of the gene encoding GTP: Mannose-1-phosphate guanyltransferase, *mpg1*, increases cellular GDP-mannose levels and protein mannosylation in *Trichoderma reesei*. Appl. Environ. Microb..

[36] Farooq M., Hussain M., Nawaz A., Lee D.-J., Alghamdi S.S., Siddique K.H. (2017). Seed priming improves chilling tolerance in chickpea by modulating germination metabolism, trehalose accumulation and carbon assimilation. Plant. Physiol. Biochem..

[37] Liu X., Fu L., Qin P., Sun Y., Liu J., Wang X. (2019). Overexpression of the wheat *trehalose 6-phosphate synthase 11* gene enhances cold tolerance in *Arabidopsis thaliana*. Gene.

[38] Ramon M., De Smet I., Vandesteene L., Naudts M., Leyman B., Van Dijck P., Rolland F., Beeckman T., Thevelein J.M. (2009). Extensive expression regulation and lack of heterologous enzymatic activity of the Class II trehalose metabolism proteins from *Arabidopsis thaliana*. Plant. Cell Environ..

[39] Taji T., Ohsumi C., Iuchi S., Seki M., Kasuga M., Kobayashi M., Yamaguchi-Shinozaki K., Shinozaki K. (2002). Important roles of drought-and cold-inducible genes for galactinol synthase in stress tolerance in *Arabidopsis thaliana*. Plant J..

[40] Gangola M.P., Jaiswal S., Kannan U., Gaur P.M., Baga M., Chibbar R.N. (2016). Galactinol synthase enzyme activity influences raffinose family oligosaccharides (RFO) accumulation in developing chickpea (*Cicer arietinum* L.) seeds. Phytochemistry.

[41] Oracz K., Bouteau H.E.M., Farrant J.M., Cooper K., Belghazi M., Job C., Job D., Corbineau F., Bailly C. (2007). ROS production and protein oxidation as a novel mechanism for seed dormancy alleviation. Plant J..

[42] Møller I.M., Jensen P.E., Hansson A. (2007). Oxidative modifications to cellular components in plants. Annu. Rev. Plant Biol..

[43] Gechev T., Willekens H., Van Montagu M., Inzé D., Van Camp W., Toneva V., Minkov I. (2003). Different responses of tobacco antioxidant enzymes to light and chilling stress. J. Plant Physiol..

[44] Nambeesan S., Abuqamar S., Laluk K., Mattoo A.K., Mickelbart M.V., Ferruzzi M.G., Mengiste T., Handa A.K. (2012). Polyamines attenuate ethylene-mediated defense responses to abrogate resistance to *Botrytis cinerea* in tomato. Plant Physiol..

[45] Li Z., Peng Y., Zhang X.Q., Ma X., Huang L.K., Yan Y.H. (2014). Exogenous spermidine improves seed germination of white clover under water stress via involvement in starch metabolism, antioxidant defenses and relevant gene expression. Molecules.

[46] De Rueda P.M., Gallardo E., Bueno M., Gallardo M., Sánchez-Calle I.M., Matilla A. (1993). Content and distribution of free and bound polyamines in embryonic axes of chick-pea seeds. J. Plant Physiol..

[47] Friedman R.A., Altman A., Bachrach U. (1982). Polyamines and root formation in mung bean hypocotyl cuttings: I. effects of exogenous compounds and changes in endogenous polyamine content. Plant Physiol..

[48] Huang Y., Lin C., He F., Li Z., Guan Y., Hu Q., Hu J. (2017). Exogenous spermidine improves seed germination of sweet corn via involvement in phytohormone interactions, H_2_O_2_ and relevant gene expression. BMC Plant Biol..

[49] Wang R., Gao M., Ji S., Wang S., Meng Y., Zhou Z. (2016). Carbon allocation, osmotic adjustment, antioxidant capacity and growth in cotton under long-term soil drought during flowering and boll-forming period. Plant. Physiol. Biochem..

[50] Hu W., Lv X., Yang J., Chen B., Zhao W., Meng Y., Wang Y., Zhou Z., Oosterhuis D.M. (2016). Effects of potassium deficiency on antioxidant metabolism related to leaf senescence in cotton (*Gossypium hirsutum* L.). Field Crop. Res..

[51] Wang L., Hu W., Zahoor R., Yang X., Wang Y., Zhou Z., Meng Y. (2019). Cool temperature caused by late planting affects seed vigor via altering kernel biomass and antioxidant metabolism in cotton (*Gossypium hirsutum* L.). Field Crop. Res..

[52] Nouairi I., Jalali K., Essid S., Zribi K., Mhadhbi H. (2019). Alleviation of cadmium-induced genotoxicity and cytotoxicity by calcium chloride in faba bean (*Vicia faba* L. var. minor) roots. Physiol. Mol. Biol. Plant.

[53] Lu P., Magwanga R.O., Kirungu J.N., Dong Q., Cai X., Zhou Z., Wang X., Xu Y., Hou Y., Peng R. (2019). Genome-wide analysis of the cotton G-coupled receptor proteins (GPCR) and functional analysis of *GTOM1*, a novel cotton *GPCR* gene under drought and cold stress. BMC Genomics.

[54] Hasanuzzaman M., Nahar K., Alam M.M., Bhuyan M.B., Oku H., Fujita M. (2018). Exogenous nitric oxide pretreatment protects *Brassica napus* L. seedlings from paraquat toxicity through the modulation of antioxidant defense and glyoxalase systems. Plant. Physiol. Biochem..

[55] Wang X., Yu C., Liu Y., Yang L., Li Y., Yao W., Cai Y., Yan X., Li S., Cai Y. (2019). *GmFAD3A*, a ω-3 fatty acid desaturase gene, enhances cold tolerance and seed germination rate under low temperature in rice. Int. J. Mol. Sci..

[56] Graeber K., Nakabayashi K., Miatton E., Leubner-Metzger G., Soppe W.J. (2012). Molecular mechanisms of seed dormancy. Plant. Cell Environ..

[57] Miransari M., Smith D. (2014). Plant hormones and seed germination. Environ. Exp. Bot..

[58] Bielach A., Hrtyan M., Tognetti V.B. (2017). Plants under stress: Involvement of auxin and cytokinin. Int. J. Mol. Sci..

[59] Rapp R.A., Udall J.A., Wendel J.F. (2009). Genomic expression dominance in allopolyploids. BMC Biol..

[60] Debeaujon I., Koornneef M. (2000). Gibberellin requirement for Arabidopsis seed germination is determined both by testa characteristics and embryonic abscisic acid. Plant. Physiol..

[61] Verslues P.E., Zhu J.K. (2007). New developments in abscisic acid perception and metabolism. Curr. Opin. Plant Biol..

[62] Shu K., Liu X.-D., Xie Q., He Z.H. (2016). Two faces of one seed: Hormonal regulation of dormancy and germination. Mol. Plant.

[63] Zhang T., Hu Y., Jiang W., Fang L., Guan X., Chen J., Zhang J., Saski C.A., Scheffler B.E., Stelly D.M. (2015). Sequencing of allotetraploid cotton (*Gossypium hirsutum* L. acc. TM-1) provides a resource for fiber improvement. Nat. Biotechnol..

[64] Pan X., Welti R., Wang X. (2010). Quantitative analysis of major plant hormones in crude plant extracts by high-performance liquid chromatography-mass spectrometry. Nat. Protoc..

